# Epidemiological characteristics, clinical outcomes and management patterns of metastatic breast cancer patients in routine clinical care settings of Greece: Results from the EMERGE multicenter retrospective chart review study

**DOI:** 10.1186/s12885-019-5301-5

**Published:** 2019-01-18

**Authors:** Athanasios Kotsakis, Alexandros Ardavanis, Georgios Koumakis, Epameinondas Samantas, Amanta Psyrri, Christos Papadimitriou

**Affiliations:** 1 Department of Medical Oncology, University General Hospital of Heraklion Crete Voutes, 711 10, Iraklio, Greece; 2 1st Department of Medical Oncology, Agios Savvas Anticancer Hospital, 171 Alexandras Av, 115 22 Athens, Greece; 3 2nd Department of Medical Oncology, Agios Savvas Anticancer Hospital, 171 Alexandras Av, 115 22 Athens, Greece; 4 3rd Department of Medical Oncology, Agii Anargiri Cancer Hospital, Kaliftaki 145, 14564 N. Kifissia, Athens, Greece; 5 Medical Oncology Unit, ATTIKON University Hospital, 1 Rimini St, 124 62 Athens, Greece; 6 Oncology Unit, 2nd Department of Surgery, Aretaieion Hospital, 76 Vas. Sofias Av, 115 28 Athens, Greece

**Keywords:** Metastatic breast cancer, Hormone receptor, Human epidermal growth factor receptor 2, Treatment patterns, Progression-free survival, Overall survival, Greece

## Abstract

**Background:**

The “EMERGE” study, aimed to capture real-life management patterns and outcomes in metastatic breast cancer (MBC) in Greece, also accounting for hormone (HR) and human epidermal growth factor receptor 2 (HER2) status.

**Methods:**

“EMERGE” was a multicenter, retrospective cohort study of adult MBC patients diagnosed between 01-Janaury-2010 and 30-June-2012, either de novo or having progressed from a non-metastatic state. Patient data, including treatment patterns and outcomes, were mainly abstracted through medical chart review.

**Results:**

386 patients were enrolled by 16 hospital-based oncologists between 12-March-2013 and 31-March-2015. The median look-back period was 29.1 months. At MBC diagnosis, 56.1% of the patients were HR^+^/HER2^−^, 16.6% HR^+^/HER2^+^, 14.5% HR^−^/HER2^−^, and 12.8% HR^−^/HER2^+^. In the first line setting, chemotherapy, targeted therapy and endocrine therapy were received by 76.7, 52.4, and 28.3% of the overall population, and by 66.5/36.2/42.0%, 80.4/80.4/28.6%, 88.4/90.7/0.0, and 95.6%/56.5/6.5% of the HR^+^/HER2^−^, HR^+^/HER2^+^, HR^−^/HER2^+^, HR^−^/HER2^−^ subpopulations, respectively. In the overall population, the disease progression incidence rate was 0.57 [95% confidence interval (CI): 0.48–0.67] per person-year; median progression-free survival (PFS) was 22.4 (95% CI: 20.4–24.7) and overall survival (OS) was 45.0 (95% CI: 40.9–55.0) months. Median PFS was 24.6 (95% CI: 21.3–27.9) in HR^+^/HER2^−^, 19.7 (95% CI: 12.9–25.9) in HR^+^/HER2^+^, 23.0 (95% CI: 16.6–29.7) in HR^−^/HER2^+^ and 18.3 (95% CI: 10.0–24.7) months in HR^−^/HER2^−^ subpopulations. A multivariable Cox proportional hazards model, adjusted among other factors for age and duration of diagnosis, HR and HER2 status, demonstrated that in the overall population PFS was better among those receiving first line endocrine therapy (hazard ratio: 0.70; 95%CI: 0.51–0.95; *p* = 0.024).

**Conclusions:**

“EMERGE” demonstrates differences between HR/HER2 subtypes in clinical outcomes and divergence from evidence-based guideline recommendations for MBC management, especially as it pertains to the HR^+^/HER2^−^ patients in which chemotherapy was favored over endocrine therapy in the first line setting.

**Study registration:**

The study has been registered on the electronic Registry of Non-Interventional Studies (RNIS) posted on the website of the Hellenic Association of Pharmaceutical Companies (SFEE): https://www.dilon.sfee.gr/studiesp_d.php?meleti_id=NIS-OGR-XXX-2012/1

## Background

Breast cancer is the most frequently diagnosed cancer worldwide, conferring 523,000 deaths and 15.1 million disability-adjusted life-years in women in 2015 [1]. The estimated age-standardized incidence and mortality rate of breast cancer among females in Greece for 2012, was 58.6 and 21.0 per 100,000, respectively, thus, being the leading cause of death from cancer among Greek women [2].

The past two decades have witnessed significant advances in awareness, screening and molecular understanding of breast cancer. Nevertheless, 6–10% of all women still present with distant and 30% with regional lymph node metastases [3]. Additionally, an estimated 20–50% of women diagnosed with early stage breast cancer will eventually develop metastatic disease (MBC) [4, 5]. While the 5-year survival rate for patients diagnosed with localized disease is 98.8%, the rate drops to 85.2% among women diagnosed with regional and to 26.3% for those diagnosed with distant metastases [3]. Median overall survival (OS) in the MBC setting is 2 to 3 years [6–8]. Age at diagnosis is considered one of the main prognostic factors of survival from MBC [9]. Additionally, patient comorbidities and menopausal status, tumor histology and pathology, hormone receptor (HR) and human epidermal growth factor receptor 2 (HER2) status, sites of metastatic involvement, number of involved axillary lymph nodes and de novo metastatic disease presentation are also considered prognostic factors of survival and treatment response [9–11]. HR and HER2 are key elements guiding selection of an individualized treatment strategy. Additional factors, taken into consideration when deciding on the optimal treatment, include the length of disease-free interval since primary diagnosis, presence of visceral crisis, menopausal status, patient preference and prior treatments with special challenges posed by the development of endocrine or anti-HER2 resistance [9, 10, 12, 13].

Evidence regarding the clinical management and outcomes of MBC in Greece largely stems from registries including patients participating in clinical trials, and to a lesser extent from studies conducted in the routine care [7, 14, 15]. This retrospective cohort study aimed to provide a snapshot of MBC disease burden, clinical course and healthcare resource utilization as well as to depict the management patterns in relation to HR and HER2 status in a representative population of MBC patients treated under real life clinical conditions in Greece.

## Methods

### Study design and setting

“EMERGE” was a multicenter, national, retrospective cohort study. Patient data abstraction was mainly carried out through medical chart review, but also from databases developed and maintained by co-operative groups for their research activities. As a prerequisite, the selected databases contained an adequate number of potential candidates that met the inclusion/exclusion criteria, and captured only non-identifiable patient information including the study variables. All required information for the purposes of the study was collected using paper case report forms. Chemotherapy and endocrine therapy (ET) for MBC management have been grouped using the Anatomical Therapeutic Chemical Drug Classification dictionary by the World Health Organization Collaborating Centre for Drug Statistics Methodology ‘chemical subgroup’, while targeted therapies (TT) have been presented as ‘anti-HER2 agents’, ‘antiangiogenic agents’ and ‘mTOR inhibitor’. Supportive therapies (other than radiotherapy), such as bisphosphonates have not been captured in the context of this study.

Site selection was carried out through a documented and constructed feasibility assessment process that accounted, among others, for the expertise of the investigators in clinical study conduct, their intent to comply with the study procedures and their ability to enroll the pre-specified number of patients, as well as for the availability of patients’ complete medical records. The study was conducted in accordance with the Declaration of Helsinki and all applicable local requirements.

### Study population

According to the final study protocol eligible patients were comprised of females aged ≥18 years, diagnosed with MBC between 01-Janaury-2010 and 30-June-2012, either de novo or having progressed from a non-metastatic state. Patients with a history of concurrent or other primary malignancies (except curatively resected non-melanoma skin cancer or in situ cervical cancer) were excluded from the study.

### Study objectives and endpoints

The primary endpoint of the study was the estimation of disease progression incidence rate (IR) per patient-year, for MBC patients on first line treatment. The secondary objectives of the study were to estimate the overall survival (OS), the progression-free survival (PFS) and the time to progression (TTP) and to estimate the overall all-cause mortality and MBC-related mortality rate. Furthermore, the study aimed to record clinical and pathological characteristics of newly diagnosed MBC patients and to describe the management patterns of MBC, as well as the healthcare utilization associated with the disease, in Greece.

### Statistical methods

The disease progression IR for patients receiving first line treatment, expressed in patient-years has been calculated by dividing the number of patients with progression in the first line setting by the overall time of follow-up (i.e. the sum of first line treatment duration of each patient). The Kaplan-Meier method has been used, in order to assess the OS, PFS and TTP [16]. PFS time has been defined as the time from first line treatment onset to the first documented disease progression or death due to any cause during the patients’ overall look-back period. Differences in the OS and PFS between subgroups have been examined by the log-rank test. Hazard ratios (95% confidence interval) between subgroups of interest have been derived by univariate Cox regression analysis. Likewise, a multivariable Cox proportional hazard model has been used to examine the association of PFS time with diagnosis of MBC de novo, presence of liver and bone metastases, receipt of endocrine therapy in the first line setting, HR and HER2 status, age at MBC diagnosis and MBC duration at first line treatment onset. Mortality rate, hospitalization as well as the emergency room and hospital outpatient visit rates (per patient-year) have been calculated by dividing the number of events by the sum of person-time (in years) of patient observation since MBC diagnosis. No imputation of missing data has been performed with the exception of partial dates.

Sample size evaluation was based on the maximum acceptable margin error that has been set at less than 5%. Under this consideration, a sample size of 400 patients, with a significance level a = 0.05 and power 80%, provided a margin of error of no more than 0.049. All statistical tests were two-sided and were performed at a 0.05 significance level. Statistical analysis has been conducted using SAS® v9.3 (SAS Institute, Cary, NC).

## Results

### Patient characteristics

A total of 386 patients diagnosed with MBC between 8-January-2010 and 27-June-2012 were enrolled in the study from 12-March-2013 to 31-March-2015, by oncologists practicing in 16 hospital-based sites distributed in 4 regions of Greece. The 12 sites located in Attica enrolled 77.5% of the patients. Of the enrolled patients, 7 did not meet all inclusion criteria. The median study look-back period of the eligible population was 29.1 [interquartile range (IQR): 19.2–37.7] months.

Eligible patients (*N* = 379) had been diagnosed with MBC at a mean ± SD age of 60.4 ± 12.8 years. At MBC diagnosis, 77.6% (256/330) were postmenopausal, 81.4% (206/253) had at least one term pregnancy and 54.8% (119/217) had a positive nursing history, while 92.0% (344/374) had an ECOG PS of 0 or 1. The primary tumor was histologically classified as invasive ductal carcinoma (IDC) in 76.0% of patients, as invasive lobular carcinoma in 7.9%, and for the remaining patients the tumor had characteristics of IDC with other histological features. The primary tumor differentiation was grade I or I/II in 3.4%, grade II or III or II/III in 81.5%, while for 15.0% the grade was unknown. Τhe most common metastatic sites were the bones (207/369; 56.1%), lungs (138/369; 37.4%;) and liver (28.7%; 106/369) (Table 1).

**Table 1:** Clinical and primary tumor characteristics of the overall population at MBC diagnosis (*N* = 379)

Characteristic		N (%)
Caucasians		371 (99.2)
Median age at MBC diagnosis, years		61.2 (50.7–70.9)
BMI at MBC diagnosis (kg/m^2^), median (IQR) (*N* = 320)		27.0 (23.9–31.4)
BMI category at MBC diagnosis (N = 320)	*Underweight (BMI < 18.5 kg/m* ^*2*^ *)*	2 (0.6)
	*Normal (BMI ≥ 18.5 and BMI < 25 kg/m* ^*2*^ *)*	117 (36.6)
	*Overweight (BMI ≥ 25 and BMI < 30 kg/m* ^*2*^ *)*	102 (31.9)
	*Obese (BMI ≥ 30 kg/m* ^*2*^ *)*	99 (30.9)
Menopausal status at MBC diagnosis (*N* = 330)	*Postmenopausal*	256 (77.6)
	*Premenopausal*	66 (20.0)
	*Perimenopausal*	8 (2.5)
Use of oral contraceptives (*N* = 236)		9 (3.8)
Use of hormone replacement therapy (*N* = 238)		10 (4.2)
Family history of breast cancer (*N* = 323)		47 (14.6)
Family history of ovarian cancer (*N* = 322)		5 (1.6)
Comorbidities at MBC diagnosis^a^ (N = 379)	*Vascular disorders*	61 (16.1)
	*Cardiac disorders*	51 (13.5)
	*Endocrine disorders*	50 (13.2)
	*Psychiatric disorders*	24 (6.3)
	*Metabolism and nutrition disorders*	22 (5.8)
ECOG performance status at MBC diagnosis (*N* = 374)	*PS 0–1*	344 (92.0)
	*PS 2–3*	30 (8.0)
Primary tumor location (*N* = 376)	*Left breast*	189 (50.3)
	*Right breast*	179 (47.6)
	*Both breasts*	8 (2.1)
Primary tumor size (*N* = 301)	*Tumor size ≤ 2 cm*	110 (36.5)
	*2 cm < tumor size ≤ 5 cm*	148 (49.2)
	*Tumor size > 5 cm*	43 (14.3)
Number of metastases at MBC diagnosis, median (IQR) (*N* = 264 ^b^)		2.0 (1.0–3.0)
Distant metastatic sites (*N* = 369)^a^	*Bones*	207 (56.1)
	*Lung*	138 (37.4)
	*Liver*	106 (28.7)
	*Lymph nodes*	70 (19.0)
	*Brain*	35 (9.5)
	*Pleural effusion*	29 (7.9)

^a^Only those present in at least 5% of the population have been included

^b^Patients for whom the precise number of metastases was known

IQR, interquartile range (Q25-Q75); N, number of patients with known data for each variable

### Patient disposition based on HR/HER2 status and on de novo diagnosis or progression from an earlier stage

At the time of MBC diagnosis, nearly half of the patients (49.9%; 189/379) were HR^+^/HER2^−^, while 14.8% (56/379) were HR^+^/HER2^+^, 12.9% (49/379) triple negative, and 11.3% (43/379) HR^−^/HER2^+^. Classification could not be performed in the remaining patients (11.1%; 42/379). Additionally, 59.9% (227/379) of the eligible population had been first diagnosed at an earlier breast cancer stage (a mean ± SD of 5.2 ± 5.3 years prior to MBC diagnosis), while the remaining 40.1% (152/379) presented with de novo metastatic disease.

### MBC management patterns

Of the eligible patients (*n* = 379), 99.5% had been exposed to systemic therapy, 32.2% had received radiotherapy and 13.2% had undergone surgery for MBC management. Of the patients exposed to systemic treatment (*n* = 377), 374 had received first line therapy [median exposure 5.5 (IQR: 3.7–11.0) months], 254 proceeded to second line [median 4.4 (IQR: 2.7–7.9) months], 175 to third line [median 3.5 (IQR: 1.9–5.7) months] and 105 to fourth line treatment. Information for patients of each subpopulation per HR/HER2 status that received first line treatment and those that advanced to the second, third and beyond the third line is indicated in Table 2.

**Table 2:** Systemic treatment management patterns per HR/HER2 status

	Overall	HR^+^/HER2^−^	HR^+^/HER2^+^	HR^−^/HER2^+^	HR^−^/HER2^−^
Patients receiving first line treatment	N = 374	*N* = 188	*N* = 56	*N* = 43	*N* = 46
	n (%)	n (%)	n (%)	n (%)	n (%)
Taxanes & Antiangiogenic agents	54 (14.4)	35 (18.6)	4 (7.1)	1 (2.3)	6 (13.0)
Aromatase inhibitors only	41 (11.0)	36 (19.1)	2 (3.6)	–	1 (2.2)
Platinum compounds & Taxanes & Antiangiogenic agents	19 (5.1)	5 (2.7)	–	–	13 (28.3)
Taxanes & Anti-HER2 agents	18 (4.8)	1 (0.5)	11 (19.6)	6 (14.0)	–
Pyrimidine analogues & Anti-HER2 agents	15 (4.0)	2 (1.1)	4 (7.1)	9 (20.9)	–
Other chemotherapy & Anti-HER2 agents	9 (2.4)	–	3 (5.4)	6 (14.0)	–
Patients receiving second line treatment	*N* = 254	*N* = 125	N = 36	*N* = 35	N = 33
	n (%)	n (%)	n (%)	n (%)	n (%)
Aromatase inhibitors only	21 (8.3)	18 (14.4)	1 (2.8)	–	1 (3.0)
Anti-estrogens only	20 (7.9)	17 (13.6)	–	–	–
Aromatase inhibitors & mTOR inhibitors	20 (7.9)	17 (13.6)	–	–	2 (6.1)
Pyrimidine analogues & anti-HER2 agents	20 (7.9)	1 (0.8)	7 (19.4)	10 (28.6)	2 (6.1)
Anti-HER2 agents only	11 (4.3)	1 (0.8)	1 (2.8)	8 (22.9)	1 (3.0)
Other chemotherapy & Anti-HER2 agents	6 (2.4)	–	2 (5.6)	4 (11.4)	–
Platinum compounds & Pyrimidine analogues	6 (2.4)	1 (0.8)	–	–	4 (12.1)
Aromatase inhibitors & Anti-HER2 agents	6 (2.4)	–	6 (16.7)	–	–
Patients receiving third line treatment	*N* = 175	*N* = 89	*N* = 28	*N* = 24	N = 18
	n (%)	n (%)	n (%)	n (%)	n (%)
Taxanes only	15 (8.6)	12 (13.5)	1 (3.6)	1 (4.2)	1 (5.6)
Pyrimidine analogues & Anti-HER2 agents	13 (7.4)	–	4 (14.3)	8 (33.3)	1 (5.6)
Pyrimidine analogues only	11 (6.3)	10 (11.2)	1 (3.6)	–	–
Aromatase inhibitors only	11 (6.3)	10 (11.2)	–	–	–
Anthracyclines & Other chemotherapy	7 (4.0)	4 (4.5)	–	1 (4.2)	2 (11.1)
Other chemotherapy & Anti-HER2 agents	6 (3.4)	1 (1.1)	3 (10.7)	1 (4.2)	1 (5.6)
Pyrimidine analogues & Antiangiogenic agents	5 (2.9)	2 (2.2)	–	–	3 (16.7)
Anti-HER2 agents only	5 (2.9)	–	–	5 (20.8)	–
Patients receiving beyond the third line treatment	*N* = 105	*N* = 49	N = 17	N = 18	*N* = 11
	n (%)	n (%)	n (%)	n (%)	n (%)
Pyrimidine analogues only	6 (5.7)	5 (10.2)	1 (5.9)	–	–
Anti-estrogens only	5 (4.8)	5 (10.2)	–	–	–
Anthracyclines & Anti-HER2 agents	3 (2.9)	–	–	2 (11.1)	1 (9.1)
Other chemotherapy & Anti-HER2 agents	3 (2.9)	–	–	3 (16.7)	–
Pyrimidine analogues & Anti-HER2 agents	2 (1.9)	–	–	2 (11.1)	–
Pyrimidine analogues & Other chemotherapy & Anti-HER2 agents	2 (1.9)	–	–	2 (11.1)	–
Pyrimidine analogues & Taxanes & Other chemotherapy	2 (1.9)	–	–	–	2 (18.2)

Only systemic treatment patterns utilized in at least 10.0% of one or more of the four subpopulations per HR/HER2 status are shown. Taxanes (docetaxel, paclitaxel, nab-paclitaxel); platinum compounds (carboplatin, cisplatin); pyrimidine analogues (capecitabine, fluorouracil, gemcitabine); anthracyclines (doxorubicin, liposomal and pegylated doxorubicin, epirubicin); aromatase inhibitors (anastrozole, exemestane, letrozole); anti-estrogens (tamoxifen, fulvestrant); other chemotherapy (cyclophosphamide, eribulin, methotrexate, vinorelbine); anti-HER2 agents (trastuzumab, lapatinib); antiangiogenic agents (bevacizumab, sorafenib), mTOR inhibitor (everolimus). N, number of patients analyzed for each outcome; n, number of patients who received the particular treatment

The median time elapsed from MBC diagnosis to first line treatment onset was 0.6 (IQR: 0.2–1.4) months (373 patients with available data). Chemotherapy was administered in 76.7% (287/374) of the overall population in the first line setting; TT (i.e. anti-HER2 agents, antiangiogenic agents and mTOR inhibitor) in 52.4% (196/374) and ET in 28.3% (106/374); additionally, chemotherapy, TT and ET were administered in 65.0% (165/254), 46.5% (118/254) and 36.6% (93/254) of patients in the second line; in 73.1% (128/175), 42.3% (74/175) and 28.6% (50/175) of patients in the third line setting; and in 87.6% (92/105), 42.9% (45/105), 36.2% (38/105) of patients receiving systemic fourth line treatment and beyond.

**Fig. 1:**
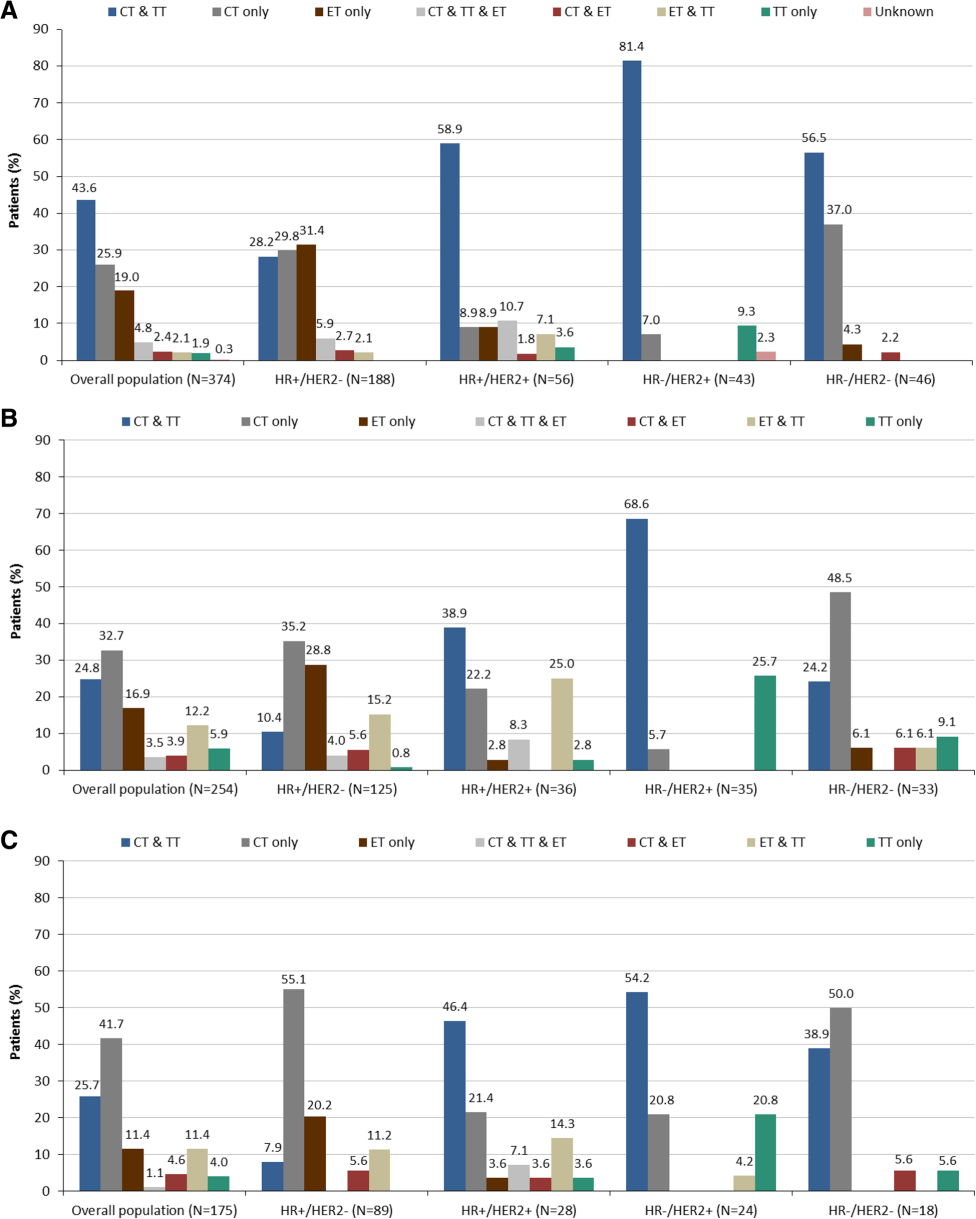
Treatment patterns of systemic therapy in the overall population and subpopulations per HR/HER2 status. Treatment patterns of chemotherapy (CT), endocrine therapy (ET) and targeted therapy (TT) utilized in the (**a**) first (**b**) second and (**c**) third line setting among patients of the overall population and in the subpopulations per HR/HER2 status

Systemic treatment patterns in terms of therapeutic drug classes administered in the first, second and third line setting of the overall population and subpopulations per HR/HER2 status are displayed in Fig. 1. Similarly, first, second, third and beyond the third line treatment patterns per therapeutic agent(s) subgroup administered in at least 10% of any of the subpopulations per HR/HER2 status are displayed in Table 2.

In regards to the treatment patterns (as displayed in Fig. 1) utilized from the first to the second and third line of therapy in the overall population, no single dominant pattern emerged. It shall be noted that a total of 30.2% (57/189), 25.0% (14/56), 58.1% (25/43) and 34.7% (17/49) of the HR^+^/HER2^−^, HR^+^/HER2^+^, HR^−^/HER2^+^ and HR^−^/HER2^−^ patients had received radiotherapy in the MBC setting.

### Clinical outcomes: Disease progression incidence rate, progression-free survival, overall survival and mortality rate

The disease progression IR in the overall population over a cumulative 256.7 years of follow-up in the first line setting, was 0.57 (95% CI: 0.48–0.67) per person-year. The IR was the lowest among HR^+^/HER2^−^ (0.47; 95% CI: 0.37–0.58) patients. Additionally, the IR was higher among patients primarily diagnosed at an earlier stage (0.85; 95% CI: 0.69–1.01) rather than de novo metastatic (0.29; 95% CI: 0.20–0.38) (Table 3).

**Table 3:** Disease progression incidence rate, PFS time and OS time

	Disease progression incidence rate				Kaplan-Meir estimated progression-free survival				Kaplan-Meier-estimated overall survival			
	N	Patients with disease progression assessment	Total follow up time in first line treatment (years)	Incidence Rate (95% CI) (person-years)	N	12-month PFS	Median PFS (95% CI) (months)	Hazard Ratio (95% CI); *p*-value, univariate analysis	N	12-month survival rate	Median OS(95% CI) (months)	Hazard Ratio (95% CI); *p*-value; univariate analysis
Overall patient population												
	357	147	256.7	0.57 (0.48–0.67)	373	76.0 (71.2–80.1)	22.4 (20.4–24.7)	n/a	379	88.8 (85.1–91.6)	45.0 (40.9–55.0)	n/a
Patient subpopulations per MBC diagnosis state												
First diagnosed at an earlier stage	208	110	129.1	0.85 (0.69–1.01)	221	68.1 (59.7–75.1)	20.4 (17.8–22.8)	1.42 (1.12–1.81); ***p*** **= 0.004**	227	87.9 (82.9–91.6)	41.0 (36.9–48.4)	1.37 (0.97–1.93); ***p*** **= 0.073**
de novo Diagnosis	149	37	127.6	0.29 (0.20–0.38)	152	82.7 (75.5–88.0)	26.8 (21.8–30.3)		152	90.1 (84.1–93.9)	not reached	
Patient subpopulations per HR/HER2 status												
HR^+^/HER2^−^	182	76	161.0	0.47 (0.37–0.58)	187	79.2 (72.5–84.5)	24.6 (21.3–27.9)	HR^+^/HER2^+^ vs. HR^+^/HER2^−^ 1.48 (1.05–2.09); ***p*** **= 0.027** HR^−^/HER2^+^ vs. HR^+^/HER2^−^ 1.21 (0.82–1.77); *p* = 0.337 HR^−^/HER^−^ vs. HR^+^/HER2^−^ 1.52 (1.06–2.16); ***p*** **= 0.023**	189	94.1 (89.6–96.7)	48.4 (36.3–61.0)	HR^+^/HER2^+^ vs. HR^+^/HER2^−^1.00 (0.60–1.68); *p* = 0.992 HR^−^/HER2^+^ vs. HR^+^/HER2^−^1.71 (1.05–2.78); ***p = 0.032*** HR^−^/HER^−^ vs. HR^+^/HER2^−^1.79 (1.12–2.87); ***p = 0.015***
HR^+^/HER2^+^	51	21	29.7	0.71 (0.40–1.01)	55	67.5 (53.0–78.4)	19.7 (12.9–25.9)		56	89.3 (77.7–95.0)	45.6 (21.5–65.3)	
HR^−^/HER2^+^	43	22	25.5	0.86 (0.50–1.22)	42	85.6 (70.8–93.3)	23.0 (16.6–29.7)		43	92.9 (79.5–97.6)	34.0 (25.5–45.6)	
HR^−^/HER2^−^	45	16	19.9	0.80 (0.41–1.20)	47	62.2 (46.3–74.6)	18.3 (10.0–24.7)		49	72.9 (58.1–83.3)	40.4 (18.4–55.0)	

Disease progression incidence rate, and the Kaplan-Meier estimated PFS time and OS time along with the 95% confidence intervals and hazard ratios of univariate analyses (*p*-values in bold indicated statistical significance) are displayed in the overall population and subpopulations per state of MBC diagnosis and HR/HER2 status. CI, confidence interval; N, number of patients analyzed for each outcome; n/a, not applicable

**Fig. 2:**
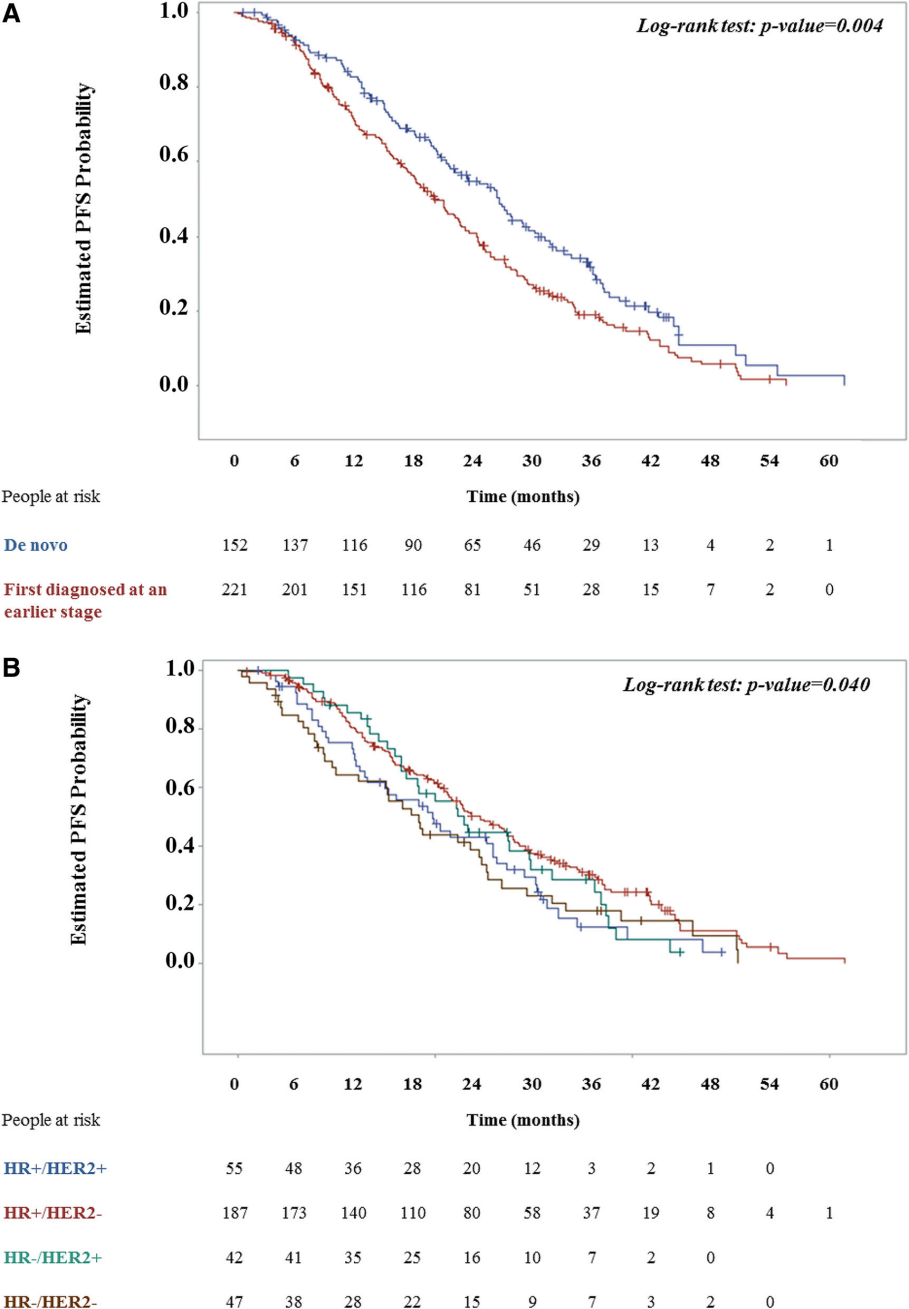
Kaplan-Meier progression-free survival (PFS) plots. PFS plots and the relevant log-rank test *p*-value is shown for MBC patients (**a**) diagnosed de novo versus at an earlier stage and (**b**) per HR/HER2 status

The Kaplan-Meier estimated median PFS of the overall population over the study observation period was 22.4 months (95% CI: 20.4–24.7) while the median TTP was 22.8 months (95% CI: 20.8–25.2). The estimated median PFS in the subpopulation primarily diagnosed at an earlier stage was shorter than that of patients diagnosed with de novo MBC (log-rank *p*-value: 0.004) (Fig. 2A and Table 3). A statistically significant difference was also detected in the median PFS of the subpopulations per HR/HER2 status (log-rank: p-value = 0.040) (Fig. 2B). Specifically, the median PFS of the HR^+^/HER2^+^ subpopulation was 19.7 months (95% CI: 12.9–25.9) and that of the HR^−^/HER2^−^ 18.3 months (95% CI: 10.0–24.7), both shorter than that of the HR^+^/HER2^−^ subpopulation (24.6 months, 95% CI: 21.3–27.9).

Among the factors (Table 4) examined by a multivariable Cox proportional hazards model in regards to their association with disease progression, median PFS was estimated to be higher among patients receiving ET as part of the first line treatment [HR: 0.70 (95% CI: 0.51–0.95; *p* = 0.024)], and lower in patients first diagnosed at an earlier breast cancer stage [HR: 1.42 (95% CI: 1.09–1.85; *p* = 0.009)] as well as among those with liver metastases [HR: 1.45 (95% CI: 1.10–1.93); p = 0.009].

**Table 4:** Multivariable Cox proportional hazard model to identify factors associated with PFS in the overall population

Multivariable Cox proportional hazard model (*N* = 319)	Hazard ratio	95% confidence interval	*p*-value
First diagnosed at an earlier stage vs. de novo MBC diagnosis	1.42^a^	1.09–1.85	0.009
Presence of liver metastases vs. No liver metastases	1.45^b^	1.10–1.93	0.009
Presence of bone metastases vs. No bone metastases	0.91^c^	0.69–1.20	0.509
Receipt of endocrine therapy vs. No endocrine therapy as part of first line treatment	0.70^d^	0.51–0.95	0.024
Negative vs. Positive HER2 status	0.91^e^	0.68–1.22	0.531
Positive vs. Negative HR status	1.01^f^	0.74–1.37	0.970
Age at MBC diagnosis (years)	1.00^g^	0.99–1.01	0.953
Time from MBC diagnosis to first line treatment (months)	0.97^h^	0.93–1.01	0.154

^a^Adjusted for presence of liver and bone metastases, receipt of endocrine therapy in the first line setting, HR and HER2 status, age at MBC diagnosis, and MBC duration at first line treatment onset

^b^Adjusted for diagnosis of MBC de novo, presence of bone metastases, receipt of endocrine therapy in the first line setting, HR and HER2 status, age at MBC diagnosis, and MBC duration at first line treatment onset

^c^Adjusted for diagnosis of MBC de novo, presence of liver metastases, receipt of endocrine therapy in the first line setting, HR and HER2 status, age at MBC diagnosis, and MBC duration at first line treatment onset

^d^Adjusted for diagnosis of MBC de novo, presence of liver and bone metastases, HR and HER2 status, age at MBC diagnosis, and MBC duration at first line treatment onset

^e^ Adjusted for diagnosis of MBC de novo, presence of liver and bone metastases, receipt of endocrine therapy in the first line setting, HR status, age at MBC diagnosis, and MBC duration at first line treatment onset

^f^Adjusted for diagnosis of MBC de novo, presence of liver and bone metastases, receipt of endocrine therapy in the first line setting, HER2 status, age at MBC diagnosis, and MBC duration at first line treatment onset

^g^Adjusted for diagnosis of MBC de novo, presence of liver and bone metastases, receipt of endocrine therapy in the first line setting, HR and HER2 status, and MBC duration at first line treatment onset

^h^Adjusted for diagnosis of MBC de novo, presence of liver and bone metastases, receipt of endocrine therapy in the first line setting, HR and HER2 status, and age at MBC diagnosis

**Fig. 3:**
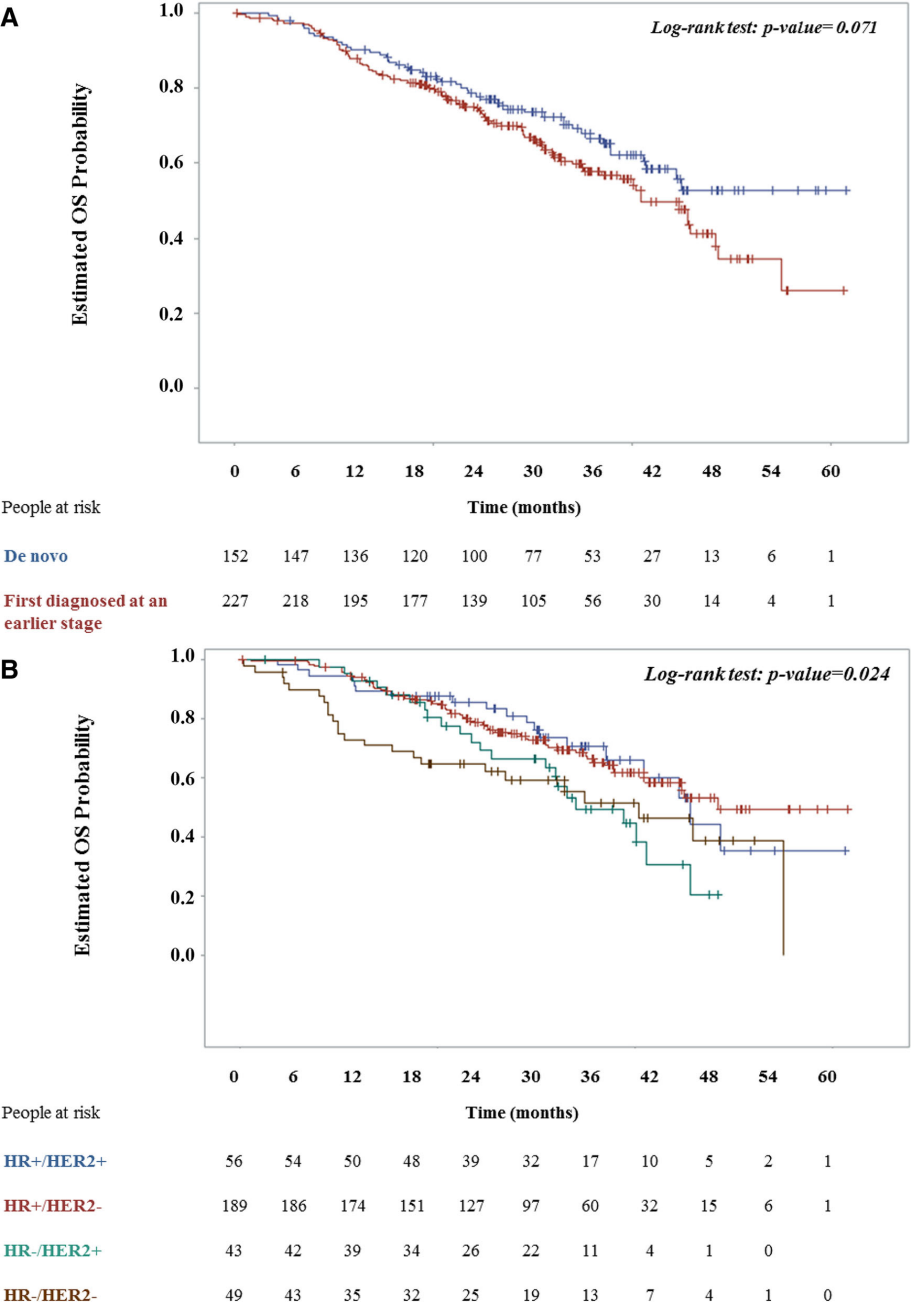
Kaplan-Meier overall survival (OS) plots. OS plots and the relevant log-rank test *p*-value is shown for MBC patients (**a**) diagnosed de novo versus at an earlier stage and (**b**) per HR/HER2 status

In regards, to the all-cause mortality rate, 143 deaths (37.7% of the overall population) due to any cause were recorded over a cumulative study observation period of 905.0 years, yielding a rate of 0.16 per person-year. The respective MBC-related mortality rate was 0.15 per person-year. The Kaplan-Meier estimated median OS was 45.0 months (95% CI: 40.9–55.0) (Table 3). The relative risk of death did not significantly differ between patients first diagnosed at an earlier breast cancer stage vs. those with a diagnosis of de novo MBC (Fig. 3A and Table 3). The relative risk of death was significantly higher for HR^−^/HER2^+^ vs. HR^+^/HER2^−^ and for triple negatives vs. HR^+^/HER2^−^ [HR: 1.71 (95% CI: 1.05–2.78; *p* = 0.032); and HR: 1.79 (95% CI: 1.12–2.87; *p* = 0.015), respectively] (Fig. 3B and Table 3).

### Healthcare resource utilization

Over a cumulative observation period of 660.5 years, the all-cause hospitalization incidence rate was 0.74 per person-year. The hospitalization incidence rate due to treatment-related toxicity and due to other reasons was 0.21 (cumulative observation period: 724.0 years) and 0.51 (cumulative observation period: 652.6 years) per person-year, respectively.

Similarly, the all-cause emergency room and hospital outpatient visit rate over a cumulative follow-up of 605.4 years was 2.67 per person-year. The emergency room and hospital outpatient visit rate for treatment-related toxicity was 0.58 (cumulative observation period: 638.1 years), while the respective rate due to other reasons was 2.02 (cumulative observation period: 618.3 years).

## Discussion

This study has yielded evidence on the clinicopathological characteristics, management patterns and treatment outcomes in the real-life clinical practice of Greece of 379 MBC patients. In addition to the overall population, treatments and clinical outcomes have been presented in the subpopulations diagnosed de novo and having progressed from a non-metastatic state and in the four subpopulations by joint HR and HER2 status. Although microarray gene-expression profiling constitutes a promising tool for prognostication and disease management, HR and HER2 status remains widely used for guiding treatment decision in the routine care [9, 10, 17, 18]. Overall, of the 337 MBC patients with known HR/HER2 status enrolled in the study, 56% were HR^+^/HER2^−^, while a nearly equal percentage were HR^+^/HER2^+^ (17%) and triple negative (15%), matching the distribution reported elsewhere [18, 19].

The disease progression IR in the overall population was 0.57 (95% CI: 0.48–0.67) per person-year; the median OS was 45.0 months, i.e. higher than the general estimates of about 2 years reported elsewhere [6–8], but similar (44 months) to that reported in a study of similar design conducted in a single institution in Germany [20]. The lowest disease progression IR and the longest median PFS and OS were observed in HR^+^/HER2^−^ patients, in alignment with other studies demonstrating a better prognosis for this breast cancer entity [5]. Despite the fact that the guideline-recommended treatment of choice for HR^+^/HER2^−^ disease is sequential ET, only 42% of the subpopulation in the present study had received ET-containing first line therapy and the remaining 58% had received chemotherapy-based regimens (with or without TT). Of further concern is the fact that herein only 54% received ET in the second line setting. Suboptimal rates of ET in HR^+^/HER2^−^ patients have also been reported from data from US databases covering the decade of 2002–2012, with 60% receiving ET in the first line, and about three quarters not receiving a second ET [21]. Collectively, these observations suggest that in the real-world practice of Greece, chemotherapy is favored over ET in HR^+^/HER2^−^ patients, despite the fact that as demonstrated in this and other studies [10, 22], ET was shown to be associated with longer PFS. The factors contributing to the administration of chemotherapy over ET cannot be inferred, since the characteristics of this subpopulation have not been examined separately, and the presence of visceral crisis, the only condition under which the guidelines recommend administration of chemotherapy [9, 10], was not collected. Clarification of the reasons for this divergence from the guidelines in the routine care of Greece would be an interesting topic for further study.

In regards to triple negative cancer, the present study also supports reports that this subpopulation exhibits the worst clinical outcomes [23, 24]. Treatment for nearly all triple negative patients was comprised of chemotherapeutic regimens (96% in the first line setting), while paradoxically about 4% in the first line setting and 6% in the second line setting were exclusively treated with ET.

Furthermore, even though most studies indicate that among patients overexpressing HER2, survival and disease-free interval is better among those with a positive versus a negative HR status [25, 26], in the present study no statistical significance in the clinical outcomes of the two groups could be established, likely due to the relatively small number of patients in these two groups. In line with the evidence-based management guidelines [10], TT was used in the majority of patients overexpressing HER2; specifically, 80% of the triple positive and 93% of the HR^−^/HER2^+^ subpopulation had received TT as part of their first line therapy.

In addition, the present study adds to the observations that patients diagnosed de novo compared to those that progressed from an earlier disease state have better clinical outcomes (PFS and OS). Importantly, the survival advantage of de novo MBC patients compared to those with recurrence from an earlier stage has been linked to a short disease-free interval (≤24 months in one study [8], and < 5 years in another [11]). Furthermore, in the present study, the most common sites of metastasis were the bones, followed by the lungs, liver and brain, in line with other reports [27, 28]. Herein, the presence of liver metastasis, but not bone metastases, was shown to be associated with a shorter PFS confirming once again the perception that liver involvement in MBC signals the end of the natural course of the disease, while, on the other hand, bone involvement points out a more indolent disease course [28].

Apart from the study limitations inherent in its retrospective design, the following limitations should be acknowledged. First, for about 11% of the patients the HR/HER2 status was unclassified, due mainly to unknown HER2 status; as a result a bias of unknown magnitude and direction may have been introduced in the patient distribution per HR/HER2 status. Second, healthcare utilization was unknown for a relatively large number of patients (approximately 21% in regards to hospitalizations and 37% for emergency room and outpatient visits). Third, recording of management patterns did not extend to the utilization of supportive therapies, such as bisphosphonates, but are restricted to radiotherapy, chemotherapy, endocrine and targeted therapies in the MBC setting. In addition, regarding PFS and TTP, it should further be noted that due to the observational retrospective nature of the study, no specific response criteria or schedule of response assessments were dictated by the protocol; therefore, tumor progression assessment may have been performed less frequently than it would be in a clinical trial setting, delaying identification of disease progression, which could have led to an upward bias of these outcomes. Notably, this inherent limitation of real-world PFS hinders comparability with data generated in other studies. Furthermore, due to the large number of censored data in the OS analysis should be taken into consideration when appraising the robustness of the estimated median OS of the overall population. In contrast, the strengths of the present study lie in its medical chart review design which aided the depiction of normal clinical practice settings under real-life conditions, and in enrollment of the study population by 16 study sites geographically distributed across four regions of Greece, which allows for variations in local standards of care to be reflected.

## Conclusions

In conclusion, “EMERGE” has yielded clinically-relevant real-world data for a diverse patient population with a disease of complex and heterogeneous biology, such as MBC. The findings clearly demonstrate differences between the subtypes per HR/HER2 status in clinical outcomes and mortality and identify areas of divergence from evidence-based guideline recommendations for MBC management, especially as it pertains to the HR^+^/HER2^−^ subpopulation. In order to achieve the maximum benefit from existing, but also from promising novel treatment options for breast cancer management, further studies that specifically examine the factors guiding the physician’s treatment decision-making in routine care, should be considered.

## Abbreviations

CI: confidence interval
ET: endocrine therapy
HER2: human epidermal growth factor receptor 2
HR: hormone receptor
IDC: invasive ductal carcinoma
IQR: interquartile range
IR: incidence rate
MBC: metastatic breast cancer
OS: overall survival
PFS: progression-free survival
TT: targeted therapy
TTP: time to progression
